# Mitral Annular Forces and Their Potential Impact on Annuloplasty Ring Selection

**DOI:** 10.3389/fcvm.2021.799994

**Published:** 2022-01-04

**Authors:** Johannes H. Jedrzejczyk, Lisa Carlson Hanse, Shadi Javadian, Søren N. Skov, J. Michael Hasenkam, Marcell J. Thørnild

**Affiliations:** ^1^Department of Cardiothoracic and Vascular Surgery, Aarhus University Hospital, Aarhus, Denmark; ^2^Department of Clinical Medicine, Aarhus University Hospital, Aarhus, Denmark; ^3^Department of Surgery, University of the Witwatersrand, Johannesburg, South Africa

**Keywords:** mitral regurgitation, mitral annuloplasty device, mitral valve repair (MV repair), mitral ring annuloplasty, mitral annular forces

## Abstract

**Objectives:** To provide an overview that describes the characteristics of a mitral annuloplasty device when treating patients with a specific type of mitral regurgitation according to Carpentier's classification of mitral regurgitation.

**Methods:** Starting with the key search term “mitral valve annuloplasty,” a literature search was performed utilising PubMed, Google Scholar, and Web of Science to identify relevant studies. A systematic approach was used to assess all publications.

**Results:** Mitral annuloplasty rings are traditionally categorised by their mechanical compliance in rigid-, semi-rigid-, and flexible rings. There is a direct correlation between remodelling capabilities and rigidity. Thus, a rigid annuloplasty ring will have the highest remodelling capability, while a flexible ring will have the lowest. Rigid- and semi-rigid rings can furthermore be divided into flat and saddled-shaped rings. Saddle-shaped rings are generally preferred over flat rings since they decrease annular and leaflet stress accumulation and provide superior leaflet coaptation. Finally, mitral annuloplasty rings can either be complete or partial.

**Conclusions:** A downsized rigid- or semi-rigid ring is advantageous when higher remodelling capabilities are required to correct dilation of the mitral annulus, as seen in type I, type IIIa, and type IIIb mitral regurgitation. In type II mitral regurgitation, a normosized flexible ring might be sufficient and allow for a more physiological repair since there is no annular dilatation, which diminishes the need for remodelling capabilities. However, mitral annuloplasty ring selection should always be based on the specific morphology in each patient.

## Introduction

Every native mitral valve apparatus component is optimised to decrease and balance the trans-mitral blood pressure's systolic forces. In the closed configuration—during left ventricular systole—the leaflets are constrained at the leaflet coaptation level due to direct attachment to the chordae tendineae. Constrain is further ensured by the papillary muscles. The tethering forces in the chordae and papillary muscles must counteract the closing force's exact amount to avoid mitral prolapse. Therefore, it is paramount that the sub-valvular apparatus resists tethering forces equal to the ventricular closing force to obtain force equilibrium (1). This implies that higher left ventricular pressures may alter this equilibrium due to a higher degree of closing forces.

Disruption or impairment of individual elements in the mitral valve apparatus may change the force equilibrium condition, interfering with valve function. For example, clinical conditions such as rupture of papillary muscles or chordae tendineae could be explained by a localised elevation of closing force relative to a non-existing tethering force. Former *in vitro* studies have shown that papillary muscle displacement increases tethering forces in the chordae tendineae and the leaflets, disturbing valve coaptation (2–4). These force discrepancies ultimately result in various degrees of functional mitral regurgitation.

**Figure 1:**
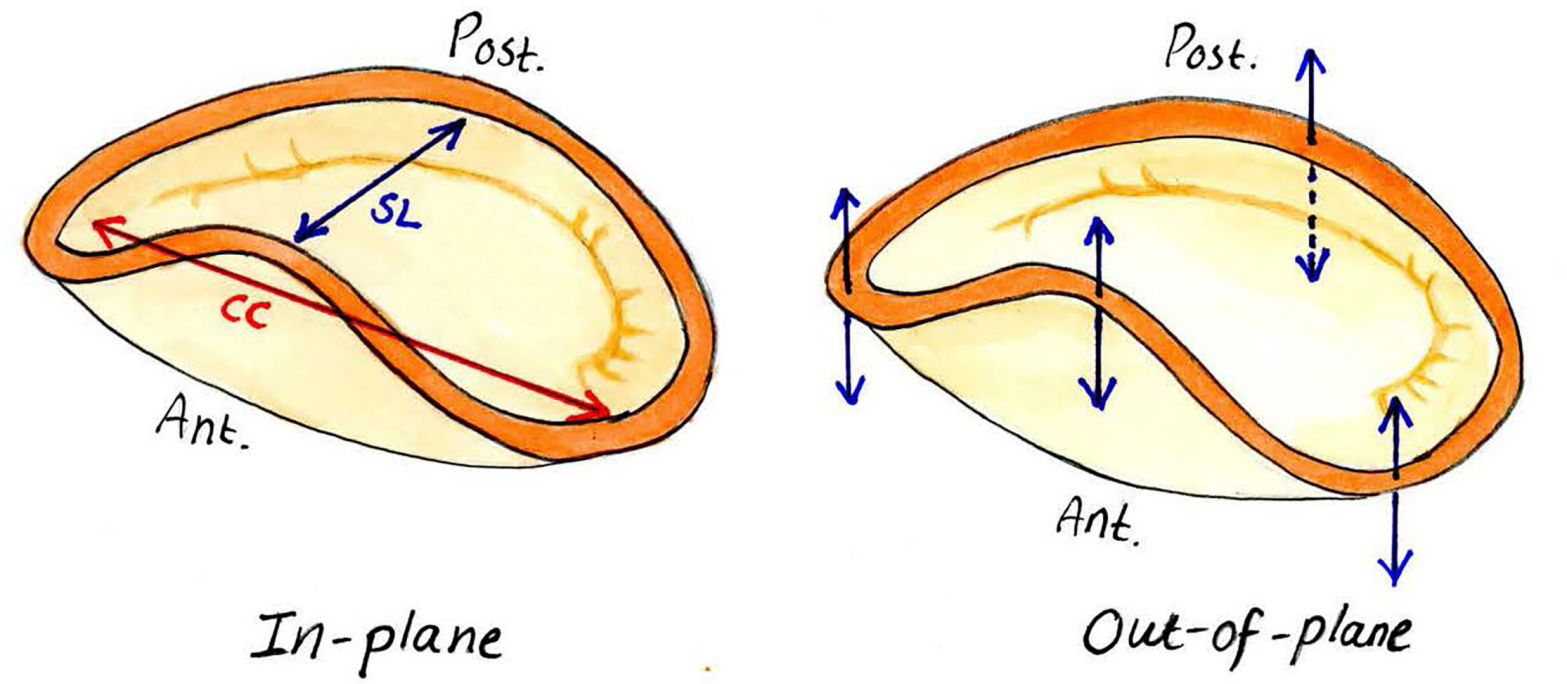
In-plane and out-of-plane forces acting on the mitral annulus. SL, septo-lateral dimension; CC, commissural-commissural dimension. The arrows represent the in-plane and out-of-plane forces, respectively.

Forces acting in the mitral annulus are often referred to and simplified as in-plane forces and out-of-plane forces. These are shown in Figure 1. The in-plane forces that act on the mitral annulus comprise deformational movements in two dimensions, which can be measured by inserting a mitral annular force transducer (5–7). For example, deformational movement from the interventricular septum to the left ventricle's lateral wall is the force acting in the septal-lateral dimension. Similarly, the deformational movement along the intercommissural line is referred to as the force acting in the commissural-commissural dimension. Finally, the forces that act in the out-of-plane direction also plays an essential role in understanding the forces that may act on mitral annular devices (6).

Surgical mitral valve repair is currently the recommended approach for severe degenerative mitral regurgitation and usually includes the implantation of an annuloplasty ring (8–11). Today, traditional annuloplasty rings available for mitral valve repair interventions are available in different shapes and different material properties. Choosing the correct type of annuloplasty ring may directly affect the patient outcome (12–14). Traditionally, mitral annuloplasty with a flexible prosthetic ring has been assumed to preserve normal mitral annular flexibility and allow a more physiological repair (15–18). Flexible rings have been thought to be advantageous since they can, in theory, change their size and shape during the cardiac cycle in the same fashion as the native annulus. However, the mitral forces are seldom considered in clinical practise as the basis for choosing an annuloplasty ring. This systematic review aims to analyse the current literature on mitral valve forces and their impact on choosing different mitral annuloplasty rings and facilitate surgeons' choice of mitral valve rings in clinical practise.

## Materials and Methods

### Study Design

This systematic review was based on the Preferred Reporting Items for Systematic Reviews and Meta-Analyses (PRISMA) guidelines (19), including a 27-item checklist to ensure correct reporting.

### Eligibility Criteria

Inclusion criteria: *In vivo* experimental setups; ovine or porcine animal models; healthy animal models; sample size ≥ 5; and full-text articles. Experimental studies performed on porcine models (20) and ovine models (21) were included since both species share similar anatomic and physiologic features as their human counterparts. Exclusion criteria *in vitro* models; disease animal models and reported conflict of interest.

### Search Strategy and Study Collection

PubMed, Google Scholar, and Web of Science were searched for relevant studies from 01 July 2019 to 26 September 2019 with language restricted to English. A systematic approach was used to assess all publications. Starting with the controlled subject heading “mitral valve,” Controlled subject heading-terminology was used to search for data on the following: “mitral annulus AND force,” “mitral annuloplasty ring AND force.” A total of 148 studies were found across PubMed, Google Scholar, and Web of Science. The full text of all relevant scientific publications describing the critical search terms was analysed in full for clinical data and endpoints. The search was expanded until no further publications were found.

### Data Collection

In each study, the following information was gathered: author, country of study, year of publication, journal of publication, study design, surgical procedure, type of force transducer, data acquisition protocol, and how data was processed.

### Study Selection and Methodological Quality

**Figure 2:**
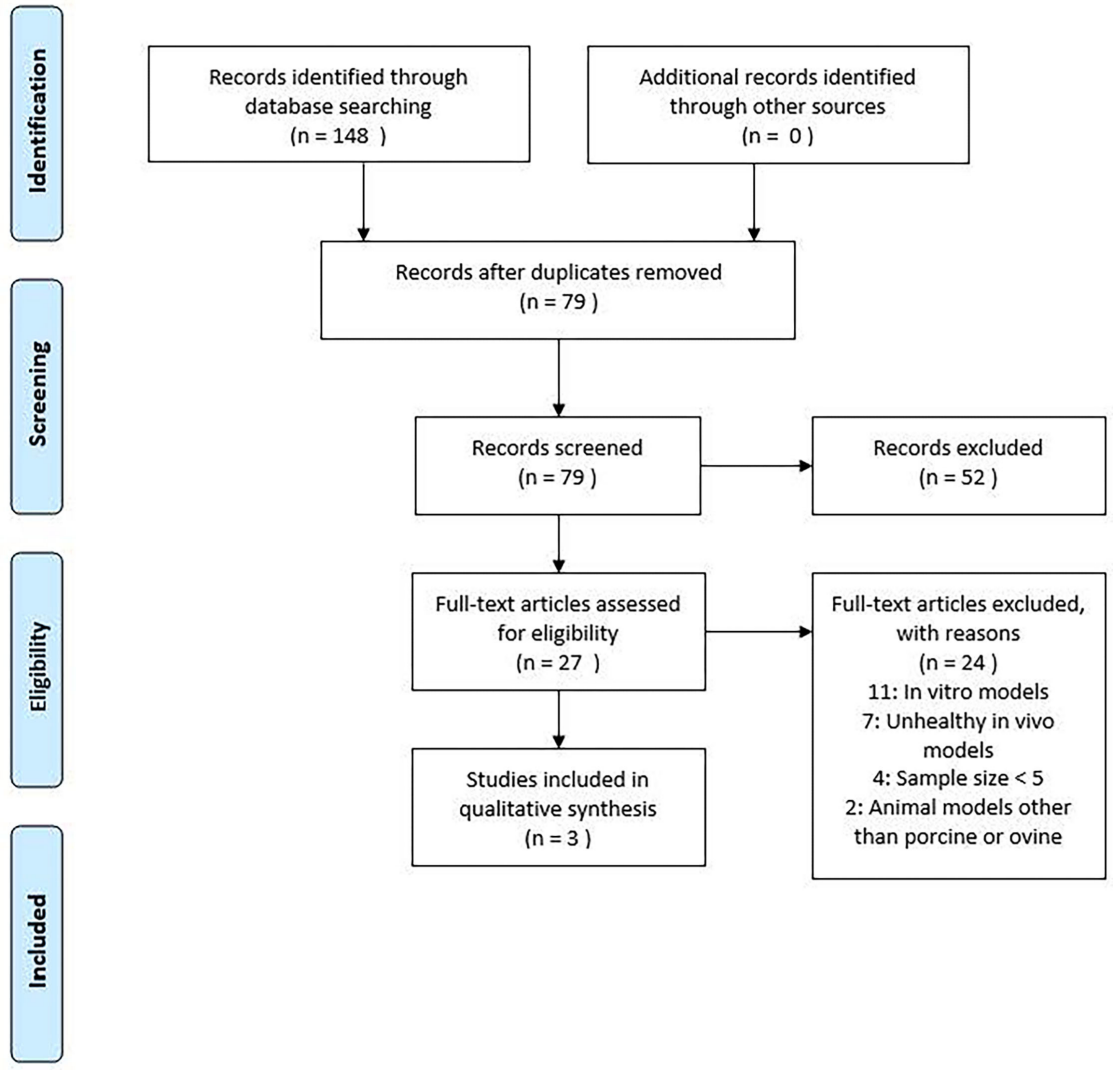
PRISMA flow chart used for study selection.

The review of publications was approached using the PRISMA flow chart (Figure 2). Titles and abstracts were screened for eligible studies, which were read in full. The scientific methodological quality of the included studies was also assessed. However, no quantitative rating tool was used since “quality assessment of observational studies is not commonplace in systematic reviews” (22). If disagreement occurred, a consensus was reached through discussion among all the authors.

## Results

### Study Characteristics

We identified 148 studies. After screening the title and abstract and removing duplicate studies, 27 were considered eligible for full-text assessment (Figure 2). Inclusion criteria were met in three studies as listed in Table 1.

**Table 1:** Characteristics of included studies.

**References**	**Design**	**Sample size**	**Animal model**	**Healthy animal model**	**Full-text article**
Siefert et al. (6)	*In vivo*	6	Ovine	Yes	Yes
Skov et al. (23)	*In vivo*	7	Porcine	Yes	Yes
Jensen et al. (24)	*In vivo*	8	Porcine	Yes	Yes

*The included studies met all eligibility criteria*.

### In-plane Forces in the Native Mitral Annulus

The first measurements of in-plane mitral valve forces were described in 1994 by Hasenkam et al. They measured a maximum force of 6–8 N to act 30° clockwise from the natural intercommissural line indicating significant force accumulations during the systolic contraction of the myocardium (25). Later, Seifert et al. (6) and Skov et al. (7, 23) developed dedicated mitral annular force transducers to describe the in-plane forces. The results are summarised in Table 2. Skov et al. hypothesised that a linear superposition of strain contribution created from two simultaneous force directions might cause misinterpretation of calibrated in-plane forces by order of magnitude of up to 20 N when an out-of-plane force of 2–3 N was present (7). To circumvent this problem, Skov et al. developed a crosstalk elimination algorithm that uses the out-of-plane forces and the linear dependency to remove the out-of-plane strain contribution from the in-plane strain measurements. Caution should be exercised when comparing absolute values between transducers with different frame designs, materials, and calibration setups. It should also be stressed that Skov et al. (7, 23) applied crosstalk elimination, while Siefert et al. (6) did not. This might explain why Skov et al. observed lower in-plane forces compared to Siefert et al. Lastly, it is crucial to consider that Seifert et al. used an ovine model, while Skov et al. used a porcine model. Nevertheless, a crude assessment would assume that the actual maximum septal-lateral force would lie between 2.1 and 5.2 N, and the actual maximum commissural-commissural force would lie between 3.0 and 3.8 N.

**Table 2:** Overview of in-plane forces in the native mitral annulus.

**References**	**Number of subjects**	**Type of study**	**Crosstalk elimination**	**Left ventricular pressure (mmHg)**	**Septal-lateral force (*N*)**	**Commissural-commissural force (*N*)**
Siefert et al. (6)	6	*In vivo*	No	125	5.2 ± 1.0	3.8 ± 1.0
Skov et al. (23)	7	*In vivo*	Yes	107 ± 25	2.1 ± 1.0	3.0 ± 1.1

*Two studies measured in-plane forces in vivo (6, 23)*.

### Out-Of-Plane Forces in the Native Mitral Annulus

Jensen et al. (5, 24) were the first to describe the distribution and importance of the out-of-plane forces on flat and saddle-shaped annuloplasty rings. Recently, Skov et al. (7) measured the out-of-plane mitral annular forces using a novel mitral annular force transducer optimised to distinguish annular segments and multiplane forces. Following crosstalk elimination, the out-of-plane annular segments experienced an average force of 1.4 ± 0.4 N in the pilot study (7). Later, Skov et al. used the same force transducer to measure out-of-plane in a more extensive study (*n* = 7) (23). The out-of-plane force measurements for the individual annular segments are summarised in Table 3.

**Table 3:** Overview of out-of-plane forces in the native mitral annulus.

**References**	**Number of subjects**	**Type of study**	**Anterior annular segment (***N***)**	**Posterior annular segment (***N***)**	**Anterior commissural segment (***N***)**	**Posterior commissural segment (***N***)**
Jensen et al. (24)	8	*In vivo*	0.64 ± 0.08	0.15 ± 0.21	−1.59 ± 0.38	−1.30 ± 0.38
Skov et al. (23)	7	*In vivo*	0.87 ± 0.53	0.51 ± 0.26	0.92 ± 0.54	0.50 ± 0.39

*Two studies measured out-of-plane forces in vivo (23, 24)*.

Skov et al. (7) concluded that the measured restraining forces indicated a systolic strain accumulation in the anterior annular segment originating from apically directed forces. This corresponds to a bending of the annular force transducer across the septal-lateral dimension. Both commissural segments experienced a similar negative systolic strain accumulation corresponding to forces directed away from the apex that flexes the device across the commissural-commissural dimension. This suggests that the annulus attempts to transform the device into an out-of-plane saddle shape. It is in agreement with previous studies (5, 24), which also conclude that the direction of the systolic out-of-plane forces indicated movements towards a saddle-shaped annulus. Accordingly, Jensen et al. concluded that “Saddle-shaped annuloplasty rings provide superior uniform annular force distribution compared to flat rings and appear to represent a configuration that minimises out-of-plane forces that could potentially be transmitted to leaflets and chords.” (24). The authors further conclude that this may have significant implications for choosing the optimal annuloplasty ring (24).

#### Mitral Annuloplasty Devices

A recent study characterised the remodelling effects and deformational forces of true-sized rigid, semi-rigid, and flexible mitral annuloplasty rings in a healthy porcine model (23). Results showed that cyclic changes in the mitral annulus circumference are significantly lower for all types of annuloplasty rings (flexible: 7 ± 3 mm, semi-rigid: 4 ± 2 mm, and rigid: 2 ± 1 mm) compared with the native mitral annulus (11 ± 5 mm). This indicates a remodelling capacity of all annuloplasty ring types. Changes in mitral annular geometry and cyclic deformation for different annuloplasty ring types (23) are summarised in Table 4.

**Table 4:** Overview of geometric changes in the mitral annulus in accordance with ring type.

	**Change in mitral annular area (mm^**2**^)**	**Change in mitral annular circumference (mm)**	**Change in septal-lateral distance (mm)**	**Change in commissural-commissural distance (mm)**
Native mitral annulus	146 ± 61	11 ± 5	4.3 ± 2.6	3.3 ± 2.7
Flexible ring	98 ± 59	7 ± 3	3.7 ± 1.5	4.2 ± 0.8
Semi-rigid ring	44 ± 16	4 ± 2	1.1 ± 0.3	1.4 ± 0.7
Rigid ring	40 ± 20	2 ± 1	1.5 ± 1.4	1.8 ± 1.0

*Cyclical changes of the mitral annular area as well as septal-lateral and commissural-commissural distances according to ring type (23)*.

Both in-plane and out-of-plane forces in the mitral annulus were measured using a mitral annular force transducer (7, 23). The force transducer was superimposed onto the annuloplasty ring to measure how much force the annuloplasty ring would absorb – thereby reflecting the remodelling capacity of the annuloplasty ring. All forces were generally lower for semi-rigid and rigid rings than the flexible rings and the native mitral valve (no ring). Mitral annular force measurements (23) are summarised in Table 5.

**Table 5:** Overview of forces in the mitral annulus in accordance with ring type.

	**Out-of-plane segments (** ***N*** **)**				**In-plane segments (** ***N*** **)**	
	**Anterior annular segment**	**Posterior annular segment**	**Anterior commissural segment**	**Posterior commissural segment**	**Septal-lateral dimension**	**Commissural-commissural dimension**
Native mitral annulus	0.87 ± 0.53	0.51 ± 0.26	0.92 ± 0.54	0.50 ± 0.39	2.12 ± 1.09	2.98 ± 1.12
Flexible ring	0.34 ± 0.19	0.51 ± 0.54	0.87 ± 0.61	0.75 ± 0.36	2.66 ± 1.82	3.43 ± 1.62
Semi-rigid ring	0.33 ± 0.20	0.58 ± 0.36	0.41 ± 0.29	0.34 ± 0.28	2.37 ± 1.51	1.35 ± 0.78
Rigid ring	0.63 ± 0.60	0.30 ± 0.15	0.41 ± 0.17	0.23 ± 0.14	1.21 ± 0.77	1.23 ± 0.79

*Forces in the mitral annulus according to ring type with forces in the native mitral annulus as reference (23)*.

Forces in the commissural-commissural dimension are significantly reduced after implantation of a semi-rigid or rigid ring (semi-rigid: 1.4 ± 0.8 N, rigid: 1.2 ± 0.8 N) compared with implantation of a flexible ring or the native heart (flexible: 3.4 ± 1.6 N, native heart: 3.0 ± 1.6 N) (23). This indicates that semi-rigid and rigid rings restrict the mitral annular dynamics equally, with no significant difference in remodelling capacity. In contrast, flexible rings do not seem to alter the mitral annular dynamics and force distribution, which implies the limited remodelling capabilities of flexible mitral annuloplasty rings (23). Furthermore, Spoor et al. found that patients with congestive heart failure who previously had a flexible mitral annuloplasty ring inserted were more prone to develop recurrent mitral regurgitation compared with patients who had received implantation of a non-flexible ring (26). This may indicate that rigid and semi-rigid annuloplasty rings have superior remodelling capabilities compared with flexible annuloplasty rings.

In a study by Caiani et al., the remodelling effects after repair of mitral valve prolapse, respectively, with a complete rigid ring (*n* = 23) and an incomplete ring (*n* = 21) was evaluated (27). Both ring types effectively reduced the annular size and diameter; only a minor increase in area was observed during systole. In addition, mitral annular height was effectively reduced with the complete and the incomplete ring type. However, the complete ring ensued a more significant reduction in annular height compared with the incomplete ring (4.6 vs. 7.2 mm *P* < 0.001) (27), thereby increasing the planarity of the mitral annulus. These findings are in accordance with the generally accepted theory that flexible rings preserve the normal mitral annular flexibility and allow for a more physiological repair (15–18). The results found by Caiani et al. (27) indicates that this is also true for incomplete flexible rings. Kwon et al. examined the management of functional ischemic mitral regurgitation with a complete ring (*n* = 209) compared with an incomplete ring (*n* = 270) in a retrospective study (28). Even though evidence suggests that patients receiving a complete mitral annuloplasty device had a greater risk of the ongoing deterioration of cardiac function, the authors found that the use of a complete mitral annuloplasty device was associated with a lower risk of recurrent moderate to severe mitral regurgitation compared to partial mitral annuloplasty devices (28). The results from Kwon et al. suggest that complete mitral annuloplasty devices possess more prominent remodelling capabilities compared to partial mitral annuloplasty devices.

During systole, the mitral valve naturally conforms to an out-of-plane saddle shape and dilates back to a more flat conformation during diastole (29–33). Salgo et al. stated that the saddle shape is conserved across mammalian species and provides indirect evidence of its advantage (31). In line with this train of thought, Jensen et al. (30) hypothesised that saddle-shaped annuloplasty rings cause less distortion of the physiologic leaflet geometry than flat rings. Furthermore, they observed that saddle-shaped annuloplasty rings maintain both leaflets operational while flat rings rendered the posterior leaflet immobile and the anterior leaflet aligned flat along the annulus in systole resulting in a “monoleaflet” functionality *in vivo*. This led Jensen et al. to conclude that saddle-shaped annuloplasty rings provide better leaflet coaptation geometry than flat rings (30). Jensen et al. (30) furthermore hypothesised that a possible mechanism for the restrictive motion of the posterior leaflet after annuloplasty could be that true-sized annuloplasty rings may relocate the posterior annulus towards the centre of the valve orifice closer to the coaptation zone while hoisting it relative to the papillary muscle tips as earlier discovered by Green et al. (34). In clinical practise, the primary cause of monoleaflet functionality is correlated to the degree of annular downsizing since aggressive downsizing will restrict the posterior leaflet's mobility and thus result in a monoleaflet functionality. Furthermore, the amount of valve tissue and placement of neo-chordae also plays a crucial role in developing monoleaflet functionality.

To achieve competency of the left atrioventricular orifice, the anterior and posterior leaflets of the mitral valve must press upon each other to form a coaptation zone along the free edge of the leaflets. The coaptation zone is paramount to secure valve competency, and this is maintained by the support of the mitral annulus, chordae tendineae, and papillary muscles. Gogoladze et al. described the coaptation depth in the native mitral valve and mitral valves affected by functional ischemic mitral regurgitation (35). They found the native coaptation depth in region A1-P1 to be 4.9 ± 1.5 mm, the coaptation depth in region A2-P2 to be 5.2 ± 1.3 mm, and the coaptation depth in region A3-P3 to be 4.9 ± 1.3 mm. These findings were based on ten patients with normally functioning mitral valves. In addition, ten patients with functional ischemic mitral regurgitation were also examined. The coaptation depths were 7.4 ± 3.0 mm in region A1-P1, 9.7 ± 3.3 mm in region A2-P2, and 8.5 ± 3.3 mm in region A3-P3 (35). Based on these results, Gogoladze et al. found a significant difference (*p* = 0.01) between the coaptation depths in a healthy mitral valve and a mitral valve affected by functional ischemic regurgitation (35).

In a later study by the same research group, they found the coaptation depth after surgical management of functional ischemic mitral regurgitation to be 6.4 ± 2.6 mm in the A1-P1 region, 6.7 ± 2.6 mm in the A2-P2 region, and 6.3 ± 2.1 mm in the A3-P3 region (36). Although non-significant (*p* = 0.08), Greenhouse et al. found that intervention with a semi-rigid annuloplasty band shows a trend towards decreased coaptation depth in patients with functional ischemic mitral regurgitation (*n* = 16). The decrease in coaptation depth will move the line of coaptation anterosuperior closer to the anterior mitral annulus. This implies that the remodelling capabilities of a semi-rigid annuloplasty ring could positively affect the coaptation depth and, thereby, the valve's competency.

Finally, ring sizing also plays a role when choosing the optimal mitral annuloplasty device. The sizing of a mitral annuloplasty ring has traditionally been tied to the aetiology of mitral regurgitation. Accordingly, the use of an appropriately sized ring has been favoured in degenerative mitral regurgitation (37), while using a downsized ring has been advocated in ischemic mitral regurgitation (12) as well as cardiomyopathy (38). In addition, although an undersized annuloplasty ring may improve leaflet coaptation, undersized annuloplasty rings have also been associated with systolic anterior motion and postoperative mitral stenosis (39, 40). This highlights the importance of ring sizing when choosing the optimal mitral annuloplasty device for the individual patient.

## Discussion

**Figure 3:**
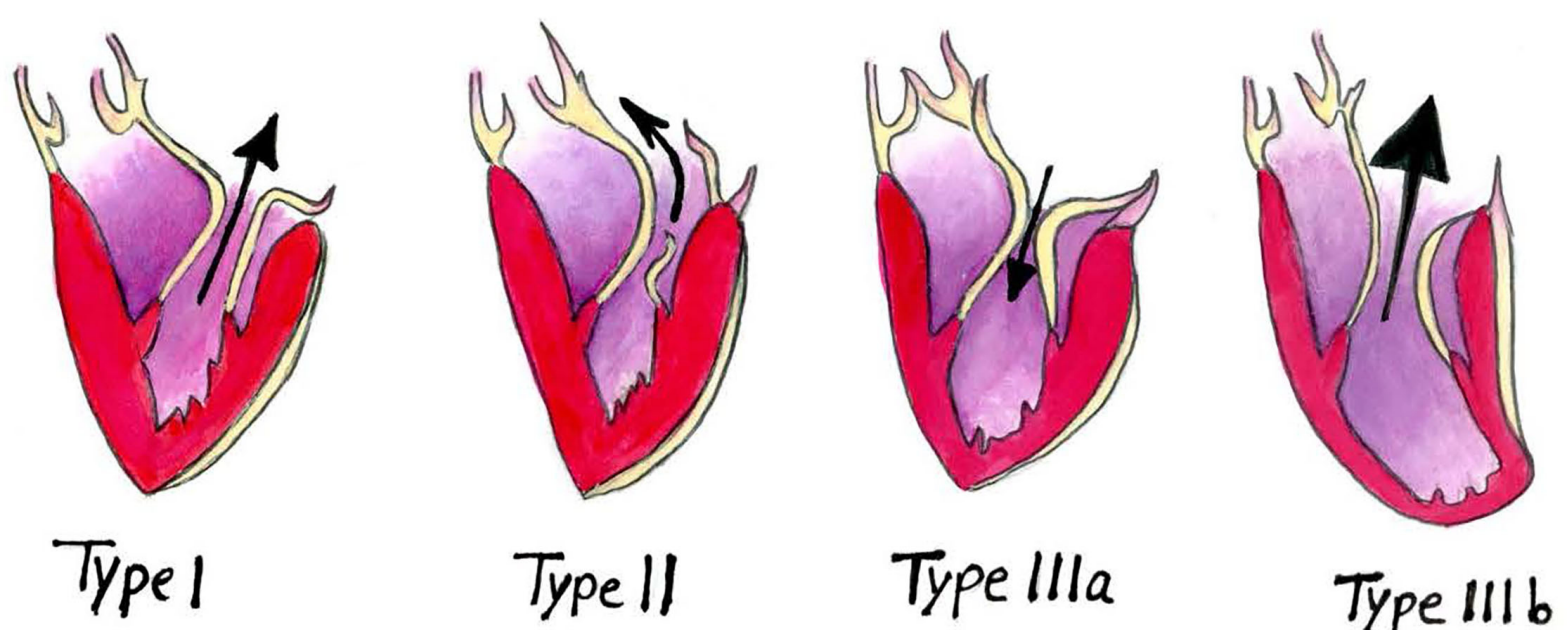
Carpentier's classification of mitral regurgitation. The arrows represent the direction of the blood flow.

The mitral valve reconstruction techniques have been well-established (41), but the choice of mitral annuloplasty ring device remains controversial. Choosing the suitable annuloplasty device has been the centre of debate and investigation to provide the surgeon with the best tools to select the individual patient's optimal annuloplasty device. However, the choice of mitral annuloplasty device still largely remains a matter of “surgeons' preference” rather than an evidence-based decision (42, 43). Based on the current literature describing force balance in the mitral valve and how it affects mitral annuloplasty rings, we have made a proposition that may aid the surgeon to improve decision making regarding the selection of mitral annuloplasty ring in each type of mitral regurgitation based on Carpentier's classification (Figure 3).

### Type I Mitral Regurgitation

Annular dilatation is the primary pathological mechanism in type I mitral regurgitation. Reducing the mitral orifice size should be the end goal, and downsizing is instrumental in achieving a successful outcome. The rigidity of the ring should be chosen depending on the severity of the annular dilatation. In severe annular dilatation cases, a rigid or semi-rigid ring would be preferable since higher remodelling capability is required to counteract the natural dilatation force (23, 26). Severe annular dilatation also abolishes the natural saddle-shaped configuration of the mitral annulus in systole. Therefore, a saddle-shaped ring might be preferable since it would help restore the native annular height to commissural width ratio (30).

A flexible ring would be preferable in patients with mild to moderate annular dilatation since lower remodelling capabilities are required. A flexible ring will, in theory, be able to change its size and shape during the cardiac cycle in the same fashion as the native annulus and may thus allow for a more physiological repair (15–18). In cases of mild annular dilatation, where the annular height to commissural width ratio is preserved, one could argue that implantation of a flat ring would be adequate. However, the literature suggests that saddle-shaped annuloplasty rings provide favourable leaflet- and chordae stress distribution while maintaining both leaflets operational (5, 7, 24, 30). Saddle-shaped rings also cause less distortion of leaflet geometry, increase the coaptation area, and decrease the risk of systolic anterior motion by increasing the anteroposterior diameter and avoiding excessive narrowing of the micro-aortic angle (44). Conversely, flat mitral annuloplasty rings render the posterior leaflet immobile, resulting in a “monoleaflet” valve formation that decreases the coaptation area. Thus, the literature seems to agree that saddle-shaped mitral annuloplasty rings are superior to flat rings in most cases. However, clinical information on a case to case basis should always be considered when choosing the ring type (6, 7).

### Type II Mitral Regurgitation

In type II mitral regurgitation, excessive leaflet motion is observed due to prolapse, chordal elongation or papillary muscle rupture. Chordal elongation is most commonly corrected by implanting artificial chords using polytetrafluorethylene sutures (Gore-Tex^®^ Suture for Chordae Tendineae, W. L. Gore & Associates, Inc.). However, a mitral annuloplasty ring is also implanted to prevent adverse events in the mitral valve apparatus. Since the mitral annulus's geometry and dynamics are unaffected in type II mitral regurgitation, a flexible mitral annuloplasty ring would be preferable. Therefore, remodelling capabilities are not required, and a flexible ring will respect the natural mitral annular dynamics to a greater extent than semi-rigid and rigid rings. Although length adjustment of Gore-Tex^®^ chords (Gore-Tex^®^ Suture for Chordae Tendineae, W. L. Gore & Associates, Inc.) is challenging and has been the source of many technical propositions, this procedure has gained increasing popularity over the years (45). One of these technical propositions features a saddled-shaped semi-rigid ring with a chordal guiding system to simplify and standardise artificial chord replacement, allowing tuning of multiple chords in less invasive approaches (46). While the chordal guiding system aids the surgeon and allows for reproducible results and reduced procedure times, an argument could be made that a flexible ring would be superior to a semi-rigid ring as the remodelling capabilities are not required in type II mitral regurgitation. The choice between a saddled-shaped ring or a flat ring is of less importance when implanting a flexible mitral annuloplasty ring as the flexible ring by nature will respect the dynamics of the mitral annulus, whether flat or saddled-shaped. However, many patients with type II mitral regurgitation simultaneously have degenerative changes in the annulus, causing it to dilate (type I mitral regurgitation). In this case, the remodelling capabilities of a semi-rigid or rigid mitral annuloplasty ring would be required.

### Type IIIa Mitral Regurgitation

Type IIIa mitral regurgitation or diastolic restricted leaflet motion is most commonly observed in patients with rheumatic valvular disease (45). The lesions encountered are most often annular dilatation, leaflet thickening, and chordal thickening or fusion. Type IIIa mitral regurgitation can be either stenotic, regurgitant or both. When annular deformation is present, or LV cavities are enlarged, a ring annuloplasty should be inserted to avoid resultant mitral regurgitation. Implantation of a rigid ring should be considered since the higher remodelling capabilities will help correct the resultant mitral regurgitation and prevent stenosis. However, mitral annuloplasty ring implantation will not correct the problem since lesions on leaflet tissue remains a concern. One approach to correct this issue is to cut the most thickened marginal chordae of the affected valve and replace them with artificial Gore-Tex^®^ chords (Gore-Tex^®^ Suture for Chordae Tendineae, W. L. Gore & Associates, Inc.) (47). Although this technique has improved long-term results, MV repair in type IIIa mitral regurgitation remains a challenge, and the rate of feasibility is still low in non-experienced centres (45).

### Type IIIb Mitral Regurgitation

Type IIIb mitral regurgitation or systolic restricted leaflet motion is primarily a ventricular disease rather than a valvular disease. It can have either an ischemic or non-ischemic origin (dilated cardiomyopathy) and is referred to as functional mitral regurgitation since the valve complex itself shows no particular anatomic lesions besides annular dilatation. A range of clinical studies has concluded that repair is associated with lower perioperative mortality and lower risk of adverse events (48–50), whereas replacement provides a better long-term correction with a lower risk of recurrence (51). Today, practise guidelines recommend treating functional mitral regurgitation with a repair procedure, although “mitral valve replacement should be considered in patients with unfavourable morphological characteristics” (9). As with type I mitral regurgitation, downsizing is paramount to repair the mitral valve successfully. An undersized mitral annuloplasty ring brings the annulus together, which consequently re-establishes the coaptation plane and reduces the central regurgitant jet created by leaflet tethering and annular dilatation. The literature seems to agree that the use of rigid- and semi-rigid annuloplasty rings is advantageous (51), which is attributable to the higher remodelling capabilities of rigid- and semi-rigid annuloplasty rings (23, 26) that prevent re-dilatation of the annulus. It might be advantageous to use a saddle-shaped ring instead of a flat ring. The saddle-shaped ring could, in theory, help restore the annular height to commissural width ratio that tends to be reduced with annular dilatation. However, this hypothesis needs further investigation.

## Conclusion

By condensing the literature on mitral valve forces and their impact on choosing different mitral annuloplasty rings, we hope to facilitate the choice of mitral valve ring in clinical practise by providing the clinician with the best tools to select the optimal mitral annuloplasty device in each type of mitral regurgitation based on Carpentier's classification.

In type I mitral regurgitation, a downsized rigid or semi-rigid saddled-shaped mitral annuloplasty ring could be advantageous as it would help correct the over-dilatation of the mitral annulus and help restore the native annular height to commissural width ratio. Correction of type II mitral regurgitation could be performed by insertion of artificial chordae along with a normosized flexible, saddled-shaped mitral annuloplasty ring to prevent adverse events in the mitral annulus. In cases of type IIIa mitral regurgitation where surgery is indicated, the most thickened marginal chords could be replaced with Gore-Tex^®^ sutures (Gore-Tex^®^ Suture for Chordae Tendineae, W. L. Gore & Associates, Inc.) and implantation of an undersized rigid or semi-rigid saddled-shaped ring. Type IIIb mitral regurgitation could be managed by implanting an undersized semi-rigid or rigid saddled-shaped mitral annuloplasty ring.

## Limitations

Force- and geometrical data presented in this review article has been collected from experimental animal studies. Thus, several inherent limitations are present, and the results should be evaluated accordingly. While the utilised animal models are highly comparable to human cardiac anatomy and physiology, subtle differences should be considered when judging the results. The animals used were all healthy without cardiac defect or disease, and one should be careful to translate the presented results directly into a clinical setting. Furthermore, all the presented experimental studies were acute, and consequently, conclusions on any long-term outcomes cannot be made.

## Data Availability Statement

The original contributions presented in the study are included in the article/supplementary materials, further inquiries can be directed to the corresponding author/s.

## Author Contributions

JJ and JH conceived and designed the study. JJ, MT, and LC collected the data and performed the analysis. SS and SJ contributed analysis tools. JJ, MT, LC, SS, SJ, and JH drafted the article or revised it critically for important intellectual content and approved the version to be published.

## Funding

The work was supported by the Novo Nordisk Foundation (Grant number NNF20OC0065584) and Medtentia.

## Conflict of Interest

Medtentia sponsored salary for performing the review but did not in any way modify or intervene in study design or its conclusions.

## Publisher's Note

All claims expressed in this article are solely those of the authors and do not necessarily represent those of their affiliated organizations, or those of the publisher, the editors and the reviewers. Any product that may be evaluated in this article, or claim that may be made by its manufacturer, is not guaranteed or endorsed by the publisher.
